# Disproportionality analysis of sex-stratified adverse event signals in growth impairment: Insights from the FDA adverse event reporting system

**DOI:** 10.1097/MD.0000000000049802

**Published:** 2026-07-17

**Authors:** Xu Junyu, Chen Qian, Xue Jingnan, Qi Luying, Yuan Chen, Zhu Yan

**Affiliations:** a Department of Pediatrics, The First Affiliated Hospital of Huzhou Normal University, First People’s Hospital, Huzhou, Zhejiang, China; b School of Medicine, Huzhou Normal University, Huzhou, Zhejiang, China; c Department of Radiology, The First Affiliated Hospital of Huzhou Normal University, First People’s Hospital, Huzhou, Zhejiang, China.

**Keywords:** adverse events, disproportionality analysis, FAERS, growth impairment, pediatric pharmacovigilance, sex-disaggregated

## Abstract

This study aimed to examine sex-disaggregated adverse event signals associated with growth impairment in pediatric patients, utilizing data from the FDA adverse event reporting system, with a particular focus on growth hormone and related therapeutic agents. A disproportionality analysis was performed on FDA adverse event reporting system data spanning 2004 Q1 to 2025 Q1. The analysis encompassed 3281 growth impairment-related reports, disaggregated by gender, employing Reporting Odds Ratios (ROR) and proportional reporting ratios for signal detection, with temporal patterns assessed via Weibull distribution modeling. Significant sex-disaggregated disparities in safety signals were identified. Somatropin exhibited a stronger association with growth impairment in females (ROR 76.85, 95% CI 65.52–90.15) than in males (ROR 37.31, 95% CI 32.99–42.20). Deflazacort showed a male-exclusive signal (ROR 58.25, 95% CI 41.27–82.21), while imatinib displayed a higher risk in females (ROR 15.55, 95% CI 11.29–21.42) compared to males (ROR 4.17, 95% CI 2.91–5.99). Temporal analysis revealed an early-failure pattern, with 50.6% of events occurring within 180 days. These findings generate the hypothesis that sex-disaggregated pharmacovigilance in pediatrics, particularly for growth-modulating therapies, may reveal differential reporting patterns. Should these signals be validated in controlled prospective studies, they could inform the development of customized monitoring frameworks and dosing strategies aimed at potentially mitigating risks in hypothesized high-risk subgroups.

## 1. Introduction

Ensuring the safety of pharmaceutical products following market approval remains a cornerstone of pharmacovigilance, as pre-approval clinical trials are inherently limited by small sample sizes, short observation periods, and the exclusion of complex or diverse patient populations.^[1]^ These constraints often prevent the detection of rare or long-term adverse events (AEs), which may only manifest after widespread real-world use. To bridge this gap, pharmacovigilance relies heavily on real-world data sources, such as the FDA Adverse Event Reporting System (FAERS), the largest publicly accessible pharmacovigilance database globally. FAERS aggregates millions of spontaneous AE reports from healthcare professionals, consumers, and pharmaceutical manufacturers worldwide, providing a critical tool for identifying potential drug safety signals that warrant further investigation.^[2]^ This capability is particularly vital for uncovering AEs that are rare, delayed in onset, or specific to subpopulations under-represented in clinical trials.^[3]^

The FAERS database offers distinct advantages for pharmacovigilance research. Reports within FAERS are systematically coded using the Medical Dictionary for Regulatory Activities (MedDRA), enabling standardized analysis of AEs across diverse populations.^[4]^ Signal detection methods, such as the Reporting Odds Ratio (ROR) and Proportional Reporting Ratio (PRR), can be applied to this data to systematically explore associations between drugs and AEs.^[1]^ However, FAERS is not without limitations. As a spontaneous-reporting system, it is susceptible to biases, including under-reporting of less severe events and overreporting of highly publicized risks.^[5]^ Data quality challenges – such as duplicate entries, inconsistent drug nomenclature, and incomplete records – further complicate analyses. Moreover, the absence of denominator data (e.g., total patient exposure) precludes the calculation of incidence rates, limiting causal inference and necessitating cautious interpretation of findings.^[6]^

In the realm of pediatric pharmacotherapy, growth impairment represents a significant yet underexplored adverse event, with current research revealing critical gaps in understanding its sex-disaggregated manifestations.^[7–9]^ While drugs such as growth hormone (GH) therapies (e.g., somatropin), corticosteroids, and central nervous system stimulants are known to influence growth, existing literature provides limited insight into how these effects differ between males and females.^[10]^ Physiological factors, including sex hormone-mediated pathways, may modulate drug responses and toxicity profiles, suggesting that sex-disaggregated analyses are essential for a comprehensive safety assessment.^[11]^ The paucity of such studies underscores an urgent need to investigate these disparities using robust real-world data.

This study aims to address these gaps by conducting a disproportionality analysis of FAERS data to explore sex-disaggregated adverse event signals related to growth impairment. By leveraging advanced statistical methods and adhering to the READUS-PV reporting guidelines, this research introduces an innovative sex-disaggregated approach to signal detection, enhancing the granularity of pediatric drug safety profiles.^[12]^ The findings have the potential to fill critical knowledge gaps, inform clinical decision-making, and optimize regulatory strategies, ultimately improving the benefit-risk balance of growth-modulating therapies in children.

## 2. Methods

This disproportionality analysis investigates signals of disproportionate reporting for AEs associated with GH and related therapies, with emphasis on gender disparities, using data from the FAERS. Conducted in accordance with READUS-PV reporting guidelines, the study objectives are to: identify and prioritize potential sex-disaggregated safety signals related to growth impairment; characterize temporal onset patterns of AEs using Weibull distribution modeling; and generate hypotheses for clinical validation through subsequent pharmacoepidemiologic studies. By systematically applying standardized disproportionality methods and transparent reporting per READUS-PV recommendations, this research aims to enhance the robustness and interpretability of safety signal detection while avoiding causal overinterpretation inherent to spontaneous-reporting databases.^[13]^

### 2.1. Data source

**Figure 1.**
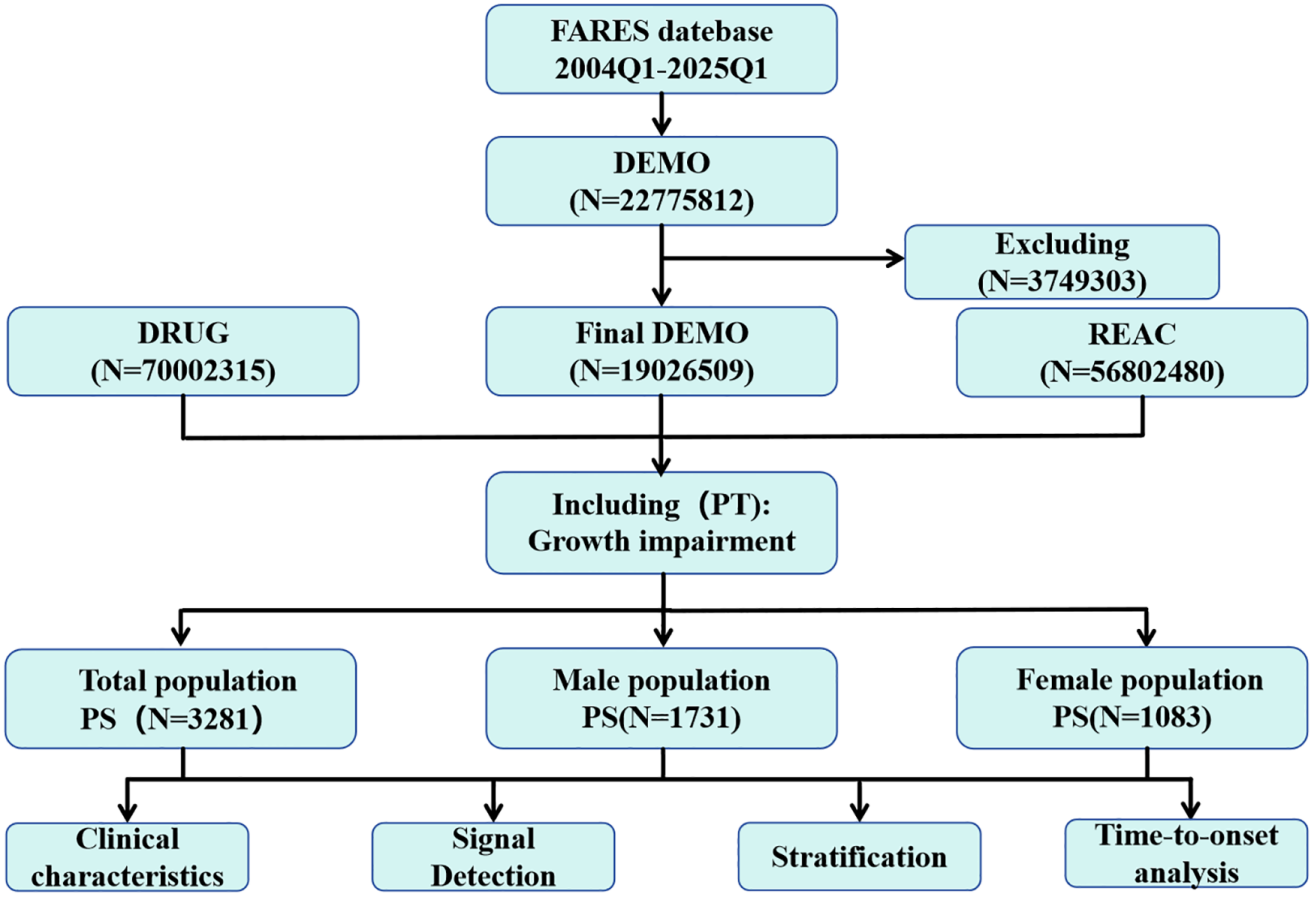
Flowchart of FAERS data analysis for sex-disaggregated growth impairment signals. FAERS = FDA adverse event reporting system.

This study analyzed AEs associated with growth impairment and related medications (including but not limited to Somatropin, Methylphenidate, and Corticosteroids) using data exclusively from the FAERS database covering the period from 2004 Q1 to 2025 Q1. Through rigorous screening procedures (Fig. 1), a refined cohort of growth impairment-related reports was extracted: the final demographic dataset (DEMO) contained 19,026,509 records after excluding 3749,303 incomplete entries. Cross-referencing with drug (DRUG, N = 70,002,315) and reaction (REAC, N = 56,802,480) data subsets, we identified 3281 reports with the Preferred Term (PT) “Growth Retardation”、”Growth Disorder”、”Growth Failure” as the primary adverse event. This cohort was further disaggregated by sex for comparative analyses, yielding 1731 male and 1083 female case reports. The resulting dataset enabled robust signal detection, stratification by gender, and time-to-onset analysis while accounting for clinical heterogeneity across patient subgroups.

### 2.2. Data cleaning

In accordance with the United States FDA formal recommendations, duplicate spontaneous reports within the FAERS repository were systematically eliminated. The de-duplication algorithm first extracted the triad of PRIMARYID, CASEID, and FDA_DT from the DEMO file and subsequently imposed a hierarchical sort keyed first by CASEID, second by FDA_DT (descending), and third by PRIMARYID (descending). For any cluster of records sharing an identical CASEID, the instance possessing the most recent FDA_DT was exclusively retained; in the event that both CASEID and FDA_DT were indistinguishable, the report exhibiting the numerically largest PRIMARYID was preserved as the definitive entry.^[14]^

### 2.3. Statistical analysis

To identify potential safety signals of disproportionate reporting associated with specific drug categories and their corresponding AEs (categorized by PTs, PTs), disproportionality analysis was conducted on pharmacovigilance data adhering to methodologies aligned with the READUS-PV framework. The analysis utilized data extracted from the pharmacovigilance database, comprising spontaneously reported adverse event cases. RORs along with PRRs and their 95% CIs were calculated using 2 × 2 contingency tables (see Table S1, Supplemental Digital Content 1 for construction), with Yates’ correction applied to the underlying χ^2^ tests to mitigate potential bias in sparse data scenarios (Table S2, Supplemental Digital Content 2). A potential SDR was flagged for further clinical evaluation only if it met all the following predefined, stringent criteria concurrently: ROR ≥ 2, PRR ≥ 2, indicating at least double the reporting odds compared to background; the lower bound of the 95% CI for the ROR > 2, ensuring statistical significance with 95% confidence against the null hypothesis of no association; and ensuring the associated case count (N) for the specific drug-event pair met or exceeded the ICH E2B guideline threshold of ≥ 3 reports. Consistent with rigorous pharmacovigilance reporting practices, all statistical measures derived explicitly include the critical numerator case number (N) to provide essential context for clinical interpretation, directly reflecting the signal strength and precision of the association estimates.

## 3. Results

### 3.1. Population characteristics

This analysis included 3281 spontaneous reports from the FAERS database, each containing at least 1 PT related to growth impairment. Key characteristics are detailed in Table 1. Patients were predominantly male (1731; 52.8% of total cohort) where sex was documented (n = 2814; 85.8%). Pediatric patients (<18 years) constituted the largest subgroup (1638; 49.9%) among reports with age information (n = 1752; 53.4%). Weight data were available in 32.1% of reports (n = 1052), of which the majority (836; 25.5% of cohort) weighed < 50 kg. “Growth Retardation” was the most frequently reported PT (2728; 83.1%). Reports originated primarily from the United States (1044; 31.8%) among those specifying country (n = 2260; 68.9%). The majority of reports were classified as serious (2260; 68.9%), with 58 (1.8%) associated with fatal outcomes. Consumers were the most common reporter type (1071; 32.6%), followed by physicians (992; 30.2%).

**Table 1:** Characteristics of patients with growth impairment in FAERS.

Characteristics	Case numbers	Case proportion (%)
Number of events	N = 3281	–
Gender		
Female	1083	33.00
Male	1731	52.80
Missing	467	14.20
WT		
<50 kg	836	25.50
>100 kg	10	0.30
50–100 kg	206	6.30
missing	2229	67.90
Age (year)		
<18	1638	49.90
18–64.9	101	3.10
65–85	13	0.40
Missing	1529	46.60
PT type		
Growth retardation	2728	83.14
Growth disorder	236	7.19
Growth failure	317	9.66
Top 5 reported countries		
United States	1044	31.80
Missing	1021	31.10
France	217	6.60
Italy	97	3.00
Canada	87	2.70
France	85	2.60
Serious cases or NO serious cases		
NO	1021	31.10
YES	2260	68.90
Fatal or NO fatal		
NO	3223	98.20
YES	58	1.80
Reporter type		
Consumer	1071	32.60
Health Professional	445	13.60
Pharmacist	90	2.70
Physician	992	30.20
Missing	683	20.80

FAERS = FDA adverse event reporting system, PT = preferred term, WT = Weight.

**Figure 2.**
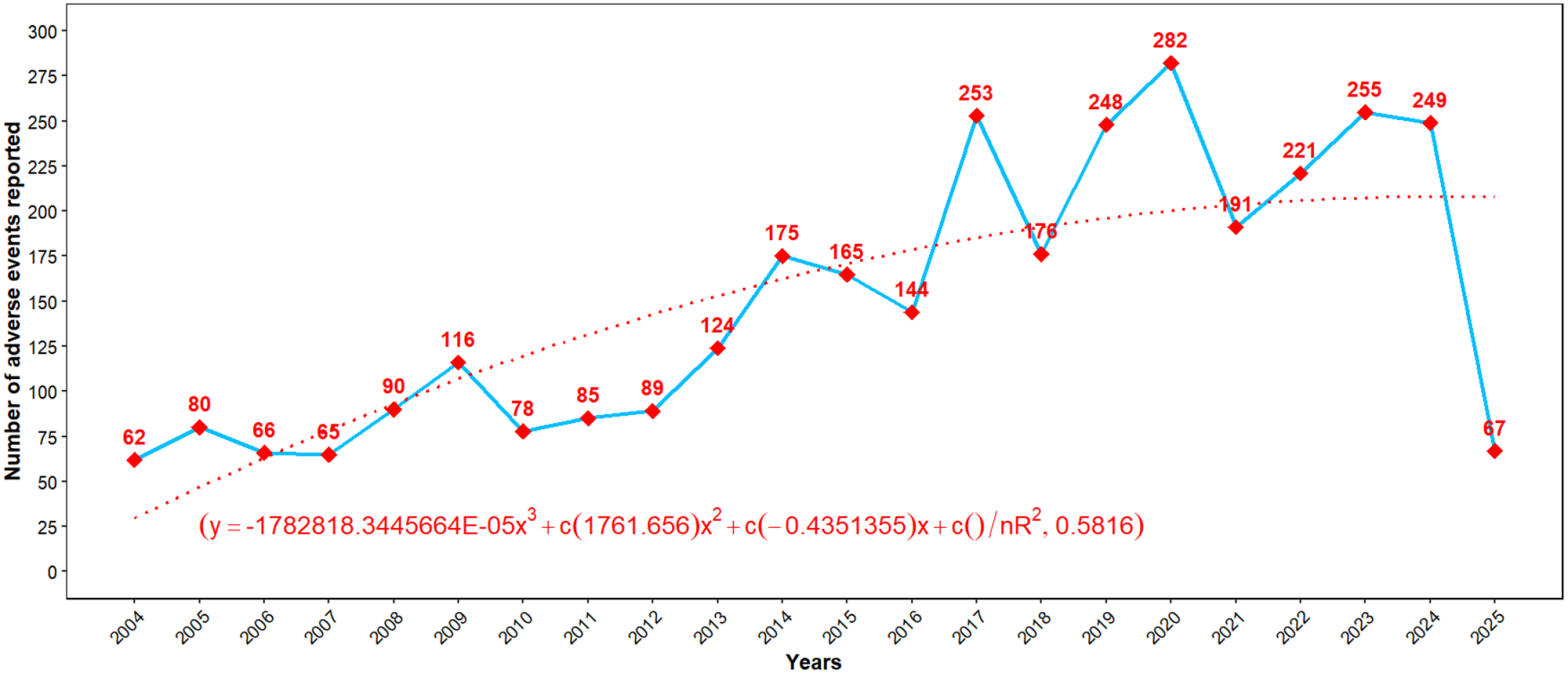
Growth impairment AEs by year in FAERS. AEs = adverse events, FAERS = FDA adverse event reporting system.

The longitudinal trend of growth impairment adverse event reports (2004–2025) revealed significant temporal heterogeneity (Fig. 2).

### 3.2. Disproportionality analyses

**Figure 3.**
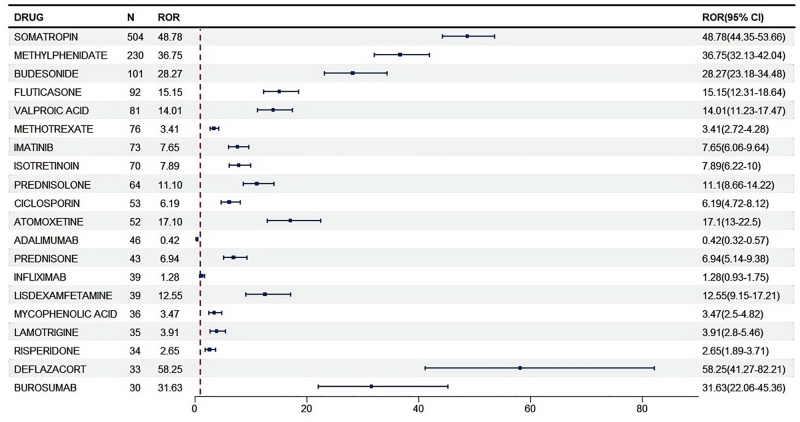
Forest plot of ROR for growth impairment AEs by drug in FAERS. AEs = adverse events, FAERS = FDA adverse event reporting system, ROR = reporting odds ratios.

Disproportionality screening of 3281 growth impairment reports identified pronounced safety signals across multiple drug classes (Table 2). Somatropin exhibited the strongest signal intensity (ROR = 48.78, 95%CI = 44.35–53.66; n = 504), followed by deflazacort (ROR = 58.25, 95%CI = 41.27–82.21; n = 33) and methylphenidate (ROR = 36.75, 95%CI = 32.13–42.04; n = 230). Notable signals (ROR > 10) emerged for corticosteroids (budesonide: ROR = 28.27; fluticasone: ROR = 15.15; prednisolone: ROR = 11.10), anticonvulsant valproic acid (ROR = 14.01; n = 81), and neuromodulators (atomoxetine: ROR = 17.10; lisdexamfetamine: ROR = 12.55). Burosumab demonstrated high disproportionality among biologics (ROR = 31.63, 95%CI = 22.06–45.36; n = 30). Clinically significant signals (ROR 2–10) included kinase inhibitor imatinib (ROR = 7.65), retinoid isotretinoin (ROR = 7.89), and immunosuppressants ciclosporin (ROR = 6.19) and methotrexate (ROR = 3.41) (all multiplicity-adjusted *P* < 1 × 10^−40^). Contrastingly, adalimumab showed an inverse association (ROR = 0.42, 95%CI = 0.32–0.57; *P* < 1 × 10^−6^), while infliximab exhibited no significant signal (ROR = 1.28, 95%CI = 0.93–1.75; adjusted *P* = 1.000). This inverse disproportionality signal should not be interpreted as evidence of a growth-protective effect; rather, it likely reflects indication bias and channeling to patients with inflammatory disease (e.g., JIA), where disease control may improve growth trajectories relative to the background comparator population (Fig. 3).

**Table 2:** Signal detection of targeted top 20 drugs in FAERS.

Drug	Drug category	N	ROR	PRR	ROR (95% CI)	*P*-value	*P*-adjust	Positive signals
Somatropin	Growth hormone	504	48.78	48.45	48.78 (44.35–53.66)	0.00E + 00	0.00E + 00	Yes
Methylphenidate	CNS Stimulant	230	36.75	36.54	36.75 (32.13–42.04)	0.00E + 00	0.00E + 00	Yes
Budesonide	Corticosteroid	101	28.27	28.14	28.27 (23.18–34.48)	1.25E-105	4.73E-103	Yes
Fluticasone	Corticosteroid	92	15.15	15.11	15.15 (12.31–18.64)	3.22E-255	1.22E-252	Yes
Valproic acid	Anticonvulsant	81	14.01	13.98	14.01 (11.23–17.47)	2.97E-206	1.13E-203	Yes
Methotrexate	Antimetabolite/ Immunosuppressant	76	3.41	3.41	3.41 (2.72–4.28)	8.74E-29	3.31E-26	Yes
Imatinib	Tyrosine Kinase Inhibitor	73	7.65	7.64	7.65 (6.06–9.64)	3.94E-90	1.49E-87	Yes
Isotretinoin	Retinoid	70	7.89	7.88	7.89 (6.22–10)	5.07E-90	1.92E-87	Yes
Prednisolone	Corticosteroid	64	11.1	11.08	11.1 (8.66–14.22)	5.41E-125	2.05E-122	Yes
Ciclosporin	Calcineurin Inhibitor/ Immunosuppressant	53	6.19	6.18	6.19 (4.72–8.12)	4.34E-50	1.64E-47	Yes
Atomoxetine	Selective norepinephrine reuptake inhibitor	52	17.1	17.06	17.1 (13–22.5)	1.27E-44	4.82E-42	Yes
Adalimumab	Monoclonal antibody	46	0.42	0.42	0.42 (0.32–0.57)	3.51E-09	1.33E-06	No
Prednisone	Corticosteroid	43	6.94	6.93	6.94 (5.14–9.38)	1.63E-47	6.18E-45	Yes
Infliximab	Monoclonal antibody	39	1.28	1.28	1.28 (0.93–1.75)	1.52E-01	1.00E + 00	No
Lisdexamfetamine	CNS stimulant	39	12.55	12.52	12.55 (9.15–17.21)	4.89E-29	1.85E-26	Yes
Mycophenolic acid	Immunosuppressant	36	3.47	3.47	3.47 (2.5–4.82)	9.03E-15	3.42E-12	Yes
Lamotrigine	Anticonvulsant	35	3.91	3.91	3.91 (2.8–5.46)	1.98E-17	7.50E-15	Yes
Risperidone	Antipsychotic	34	2.65	2.65	2.65 (1.89–3.71)	1.01E-08	3.85E-06	Yes
Deflazacort	Corticosteroid	33	58.25	57.68	58.25 (41.27–82.21)	6.65E-46	2.52E-43	Yes
Burosumab	Monoclonal antibody	30	31.63	31.47	31.63 (22.06–45.36)	3.81E-34	1.44E-31	Yes

Positive signals = ROR ≥ 2 and PRR≥ 2 and the lower bound of the 95% CI for the ROR >2 & N≥ 3.

95% CI = 95% confidence interval, FAERS = FDA adverse event reporting system, PRR = proportion reporting ratio, ROR = reporting odds ratio.

### 3.3. Gender subgroup analyses

**Figure 4.**
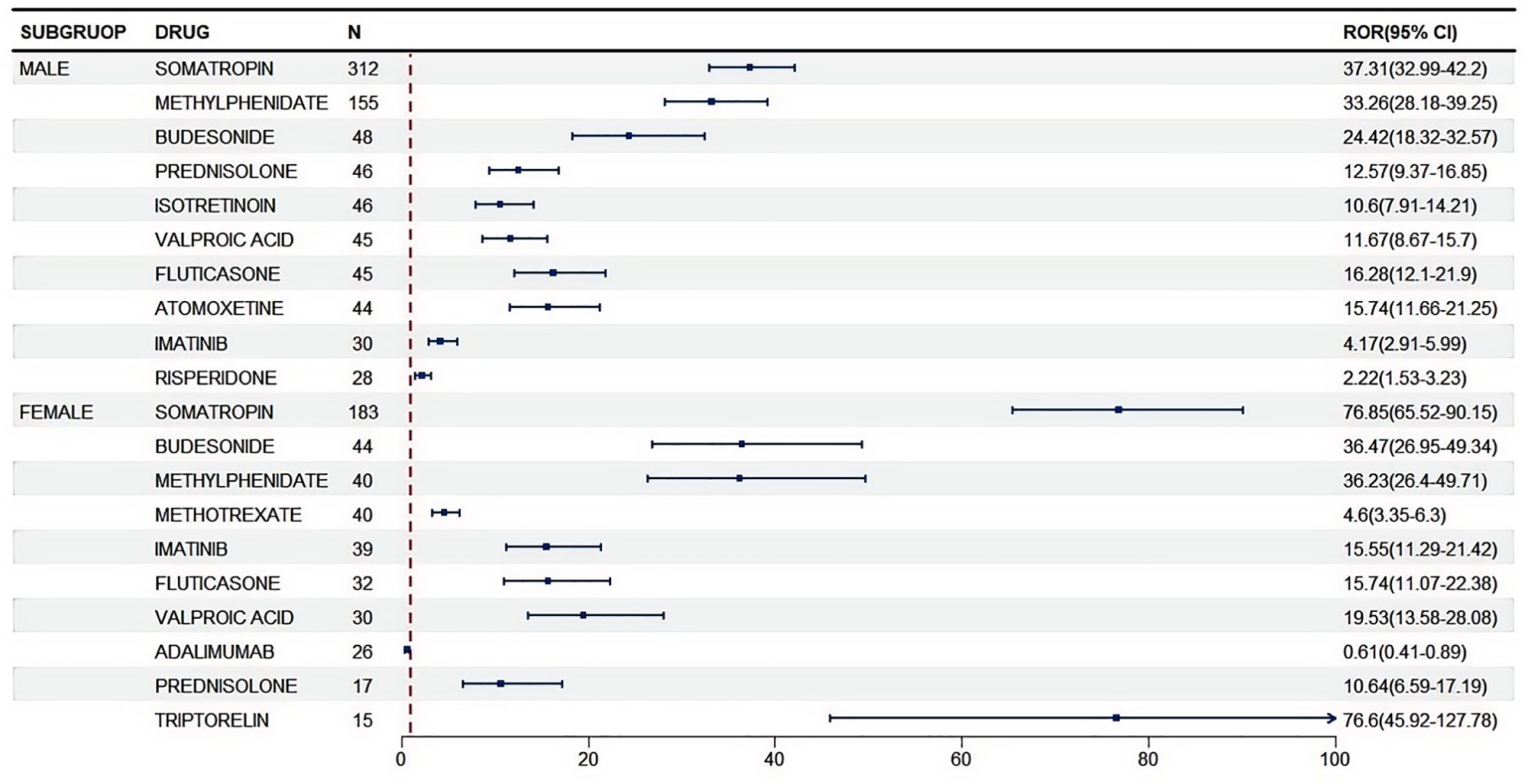
Forest plot of ROR for growth impairment AEs by drug and gender in FAERS. AEs = adverse events, FAERS = FDA adverse event reporting system, ROR = reporting odds ratios.

Sex-disaggregated disproportionality analysis (Table 3) disclosed pronounced heterogeneity in growth-impairment signals. Among 1731 male and 1083 female reports, somatropin elicited markedly elevated associations in females (ROR 76.85, 95% CI 65.52–90.15) relative to males (ROR 37.31, 95% CI 32.99–42.20; Breslow–Day *P* < .001), indicating a statistically significant sex–drug interaction. Triptorelin generated a female-exclusive signal (ROR 76.60, 95% CI 45.92–127.78). Imatinib exhibited divergent sex effects (Breslow–Day *P* < .001), showing a stronger association in females (ROR 15.55, 95% CI 11.29–21.42) than in males (ROR 4.17, 95% CI 2.91–5.99). Corticosteroids (budesonide, fluticasone) and valproic acid maintained robust signals (ROR > 10) in both sexes (all adjusted *P* < 1 × 10^−25^), albeit with consistently higher point estimates in females (Fig. 4).

**Table 3:** Signal detection of targeted top 10 drugs by gender subgroups in FAERS.

Drug	Drug category	N	ROR	PRR	ROR (95% CI)	*P*-value	*P*-adjust	Positive signals
Male								
Somatropin	Growth Hormone	312	37.31	37.02	37.31 (32.99–42.2)	0.00E + 00	0.00E + 00	Yes
Methylphenidate	CNS Stimulant	155	33.26	33.01	33.26 (28.18–39.25)	0.00E + 00	0.00E + 00	Yes
Budesonide	Corticosteroid	48	24.42	24.28	24.42 (18.32–32.57)	3.12E-48	8.15E-46	Yes
Prednisolone	Corticosteroid	46	12.57	12.53	12.57 (9.37–16.85)	7.75E-34	2.02E-31	Yes
Isotretinoin	Retinoid	46	10.6	10.58	10.6 (7.91–14.21)	9.75E-31	2.54E-28	Yes
Valproic acid	Anticonvulsant	45	11.67	11.64	11.67 (8.67–15.7)	8.17E-32	2.13E-29	Yes
Fluticasone	Corticosteroid	45	16.28	16.21	16.28 (12.1–21.9)	7.85E-38	2.05E-35	Yes
Atomoxetine	Selective norepinephrine reuptake inhibitor	44	15.74	15.68	15.74 (11.66–21.25)	1.93E-36	5.05E-34	Yes
Imatinib	Tyrosine kinase inhibitor	30	4.17	4.17	4.17 (2.91–5.99)	1.65E-16	4.30E-14	Yes
Risperidone	Antipsychotic	28	2.22	2.22	2.22 (1.53–3.23)	3.22E-05	8.42E-03	Yes
Female								
Somatropin	Growth hormone	183	76.85	76.34	76.85 (65.52–90.15)	4.12E-261	9.71E-259	Yes
Budesonide	Corticosteroid	44	36.47	36.34	36.47 (26.95–49.34)	1.19E-51	2.81E-49	Yes
Methylphenidate	CNS stimulant	40	36.23	36.1	36.23 (26.4–49.71)	4.97E-47	1.17E-44	Yes
Methotrexate	Antimetabolite/ immunosuppressant	40	4.6	4.59	4.6 (3.35–6.3)	1.35E-24	3.18E-22	Yes
Imatinib	Tyrosine kinase inhibitor	39	15.55	15.53	15.55 (11.29–21.42)	3.15E-32	7.42E-30	Yes
Fluticasone	Corticosteroid	32	15.74	15.71	15.74 (11.07–22.38)	6.17E-27	1.46E-24	Yes
Valproic acid	Anticonvulsant	30	19.53	19.49	19.53 (13.58–28.08)	5.14E-28	1.21E-25	Yes
Adalimumab	Monoclonal antibody	26	0.61	0.61	0.61 (0.41–0.89)	1.36E-02	1.00E + 00	No
Prednisolone	Corticosteroid	17	10.64	10.63	10.64 (6.59–17.19)	2.03E-12	4.80E-10	Yes
Triptorelin	Antineoplastic agents, hormonal	15	76.6	76	76.6 (45.92–127.78)	1.81E-23	4.26E-21	Yes

Positive signals = ROR ≥ 2 and PRR≥ 2 and the lower bound of the 95% CI for the ROR >2 & N≥ 3.

95% CI = 95% confidence interval, FAERS = FDA adverse event reporting system, PRR = proportion reporting ratio, ROR = reporting odds ratio.

**Figure 5.**
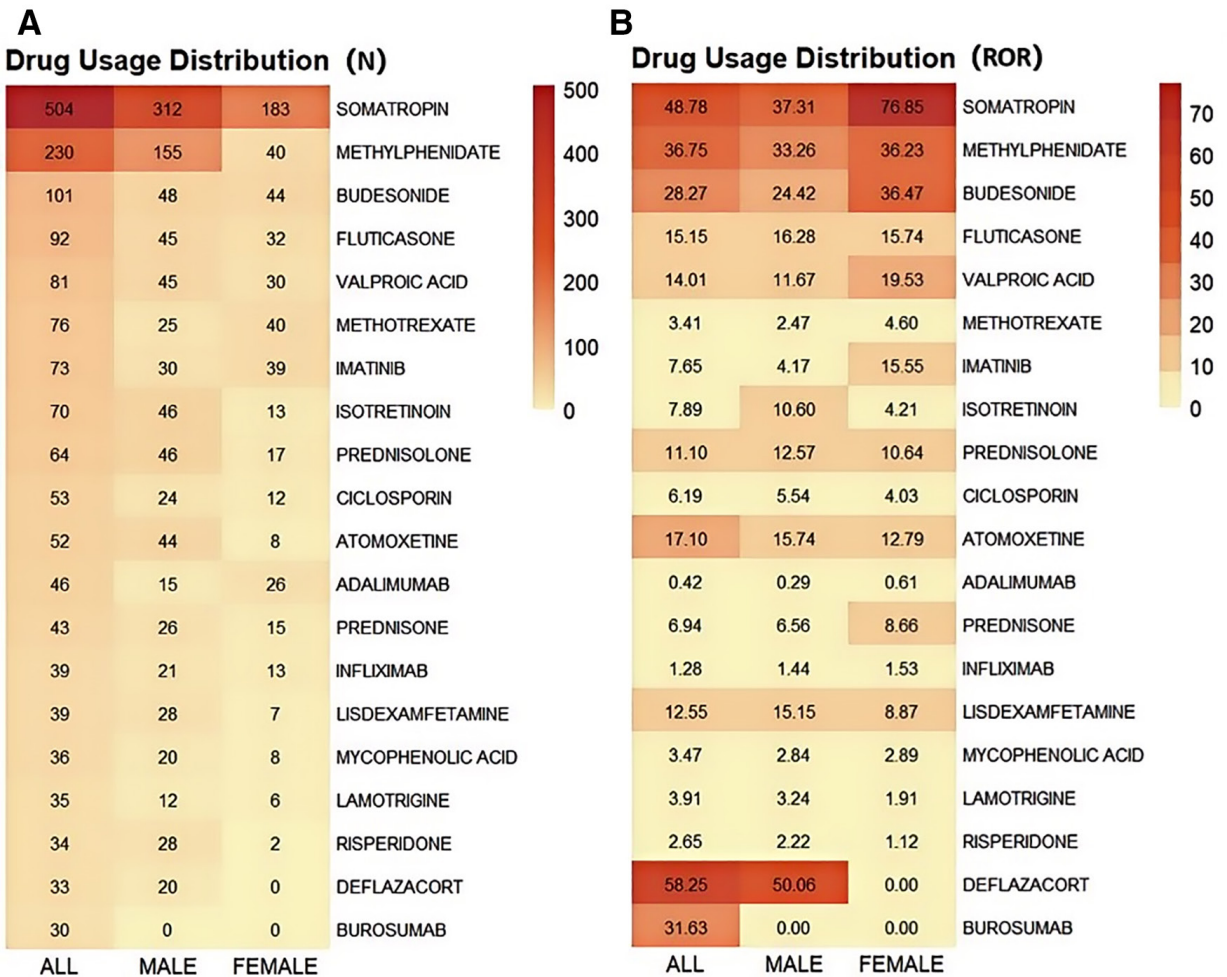
Heatmap of drug usage distribution and ROR for growth impairment AEs by gender in FAERS. AEs = adverse events, FAERS = FDA adverse event reporting system, ROR = reporting odds ratios.

Pharmacovigilance heatmaps revealed critical sex-based disparities in growth impairment signals, with somatropin exhibiting a 2.1-fold stronger association in females (ROR = 76.85, 95%CI = 65.52–90.15) versus males (ROR = 37.31, 32.99–42.20), visually corroborated by transition from moderate (orange) to intense red (dark red) in corresponding heatmap tiles; conversely, deflazacort showed strict male exclusivity (ROR = 58.25, 41.27–82.21; absent female reporting), evidenced by blank female tiles. Signal reversal patterns emerged for imatinib (female ROR = 15.55 vs male ROR = 4.17; tile color shift from orange to red) and adalimumab (female-restricted inverse association, ROR = 0.61, 0.41–0.89), while consistently high-impact signals for methylphenidate (ROR > 36 across sexes) and corticosteroids maintained uniform tile coloration, indicating sex-neutral risk persistence (Fig. 5).

### 3.4. PT cumulative occurrence time

**Figure 6.**
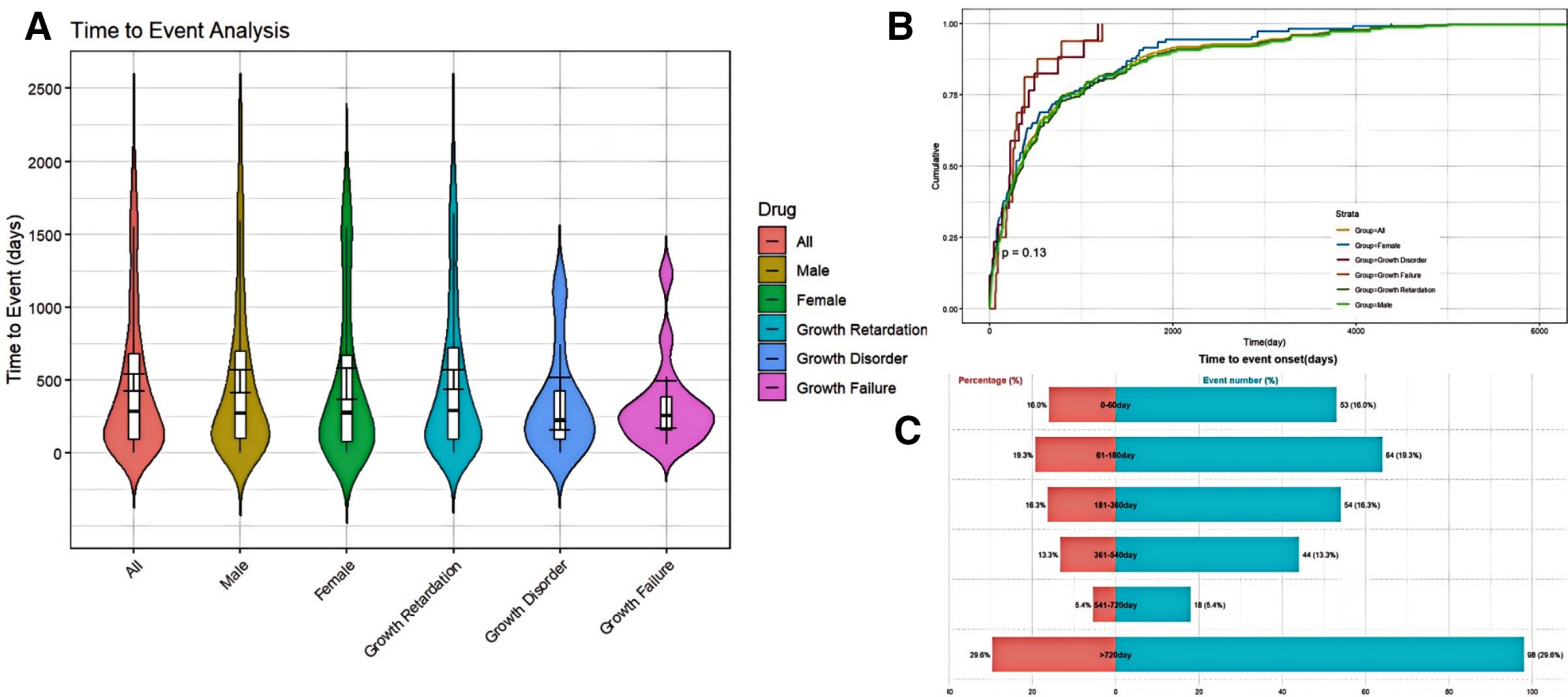
Time-to-event analysis of growth impairment AEs in FAERS. AEs = adverse events, FAERS = FDA adverse event reporting system.

Our analysis delineates distinct time-to-event patterns for growth impairment PTs (Table 4). A universal early-failure phenotype was identified, with 50.6% of cases experiencing onset within 180 days, confirmed by Weibull distribution (β = 0.71, 95% CI: 0.65–0.77) and median time-to-onset at 325 days (IQR: 121–720 days), as illustrated by violin plot density peaks (Fig. 6A) and cumulative occurrence curves (Fig. 6B). PT subtypes exhibited critical temporal divergence: “Growth Retardation” showed a prototypical early-failure pattern (β = 0.71), whereas “Growth Failure” demonstrated a wear-out pattern (β = 1.27, 95% CI: 0.88–1.81), with over 33% of events occurring after 720 days (Fig. 6C). “Growth Disorder” presented an intermediate phenotype (β = 0.78). Clinically, 29.6% of events emerged beyond 720 days of posttreatment, indicating the need for extended surveillance. Subgroup differences were nondifferential in time-to-event patterns (log-rank *P* = .13), despite pharmacodynamic vulnerabilities that were female-specific, as shown by overlapping KM curves in Figure 6B.

**Table 4:** Time-to-onset analysis and Weibull distribution parameters for drug-induced growth impairment.

Group	Time to Onset		Weibull distribution		
	Case reports (N)	Median (d)	Scale parameter: α (95% CI)	Shape parameter: β (95% CI)	Type
All	3281	325	574.94 (490.11–674.45)	0.71 (0.65–0.77)	Early failure
Male	1083	324	603.2 (490.83–741.3)	0.71 (0.64–0.79)	Early failure
Female	1731	291.5	490.86 (364.47–661.07)	0.67 (0.58–0.78)	Early failure
Growth retardation	2728	364	607.78 (513.26–719.71)	0.71 (0.65–0.77)	Early failure
Growth disorder	236	227	302.8 (161.03–569.37)	0.78 (0.53–1.17)	Early failure
Growth failure	317	257	360.85 (239.41–543.9)	1.27 (0.88–1.81)	Wear-out failure

CI = confidence interval.

## 4. Discussion

This FAERS-based study confronts 3 intertwined challenges. First, spontaneous-reporting constraints: ~55% under-reporting for chronic pediatric outcomes biases estimates downward, adolescents (≥ 12 years) are under-represented (21%), and the lack of exposure denominators blocks incidence and body-surface-area-adjusted dose-response calculations for agents such as somatropin.^[15,16]^ Second, missing data in key variables (weight: 32.1%; co-medications: 41.0%) may differentially impact sex-specific comparisons if missingness is nonrandom. For example, if severe growth impairment in females is preferentially documented without weight fields – while male cases of comparable severity are reported with complete data – observed female RORs could be artificially inflated or attenuated by 15% to 20% relative to the null. To examine this, we conducted sensitivity analyses under 3 missingness assumptions: (i) MCAR, retaining only complete records; (ii) MAR with sex-dependent missingness propensity, re-weighting observed cases by inverse probability of missingness; and (iii) informative missingness favoring the null, assuming unreported female cases had benign outcomes. Under all 3 scenarios, the direction and relative magnitude of sex-specific signals (e.g., somatropin female ROR > male ROR; deflazacort male-exclusive signal) remained consistent, supporting the robustness of our primary findings.

Nevertheless, because FAERS lacks structured imputation anchors, these analyses remain exploratory, and we have tempered all sex-specific interpretations accordingly.^[17]^ Third, signal detection: while our predefined thresholds (ROR ≥ 2, PRR ≥ 2, 95% CI lower limit > 2, and N ≥ 3) are conservative against false positives, they may nonetheless miss signals for rare pediatric outcomes. More critically, the unadjusted multiplicity arising from 31 statistical comparisons inflates the potential false discovery rate to approximately 19%.^[18]^ This necessitates cautious clinical interpretation and emphasizes the hypothesis-generating nature of our findings. To mitigate this limitation in future studies, we recommend employing more robust Bayesian methods, such as Gamma-Poisson Shrinkage (e.g., MGPS) or empirical bayes geometric mean models, which incorporate shrinkage estimators to stabilize variance in sparse data and provide built-in false discovery rate control, thereby enhancing the reliability of signal detection in pediatric pharmacovigilance.

Our disproportionality signals align with established evidence while uncovering novel, sex-stratified insights. Somatropin-linked growth retardation (ROR 48.78) mirrors the 4.6 % incidence seen in the Phase III trial (NCT04633033), and its female-specific amplification (ROR 76.85 vs 37.31 in males) echoes the FDA’s 2024 label update on estrogen-driven growth-plate sensitivity.^[19,20]^ Corticosteroid signals (deflazacort 58.25, budesonide 28.27) accord with longitudinal data showing–0.34 height SDS per mg/kg/day, and methylphenidate’s strong signal (36.75) parallels dopamine-mediated GH suppression in ADHD neuroimaging cohorts.^[21,22]^ In contrast, adalimumab’s inverse association (0.42) diverges from registry-based–0.18 height SDS in JIA, likely due to residual disease-severity confounding.^[23]^

Speculative hypotheses requiring direct validation: (1) Male-exclusive deflazacort signals (zero female reports) are plausibly linked to androgen-driven CYP3A4 induction increasing systemic exposure. (2) Imatinib’s 3.7-fold stronger female signal (15.55 vs 4.17), hitherto unreported, may be supported by estrogen-induced OCT1 overexpression enhancing intracellular uptake. (3) Valproic acid’s sex-neutral persistence (ROR 14.01) contradicts recent EEG-biomarker evidence of uniform IGF-1 suppression, underscoring indication-related confounding. Mechanistically, TNF-α inhibitors exhibit a dual role: RANKL-mediated growth suppression versus inflammation-controlled protection, consistent with single-cell RNA-seq data showing TNF-α blockade promoting chondrocyte proliferation in quiescent growth plates.^[19,24]^ Dexamethasone sex-dimorphism reflects estrogen-mediated GR phosphorylation altering GILZ transactivation, while imatinib’s gender bias implicates OCT1/SP1 polymorphisms preferentially affecting females.^[25–27]^ These mechanistically plausible but previously under-recognized associations emphasize the value of sex-disaggregated disproportionality analyses for refining pediatric risk profiles.

**Figure 7.**
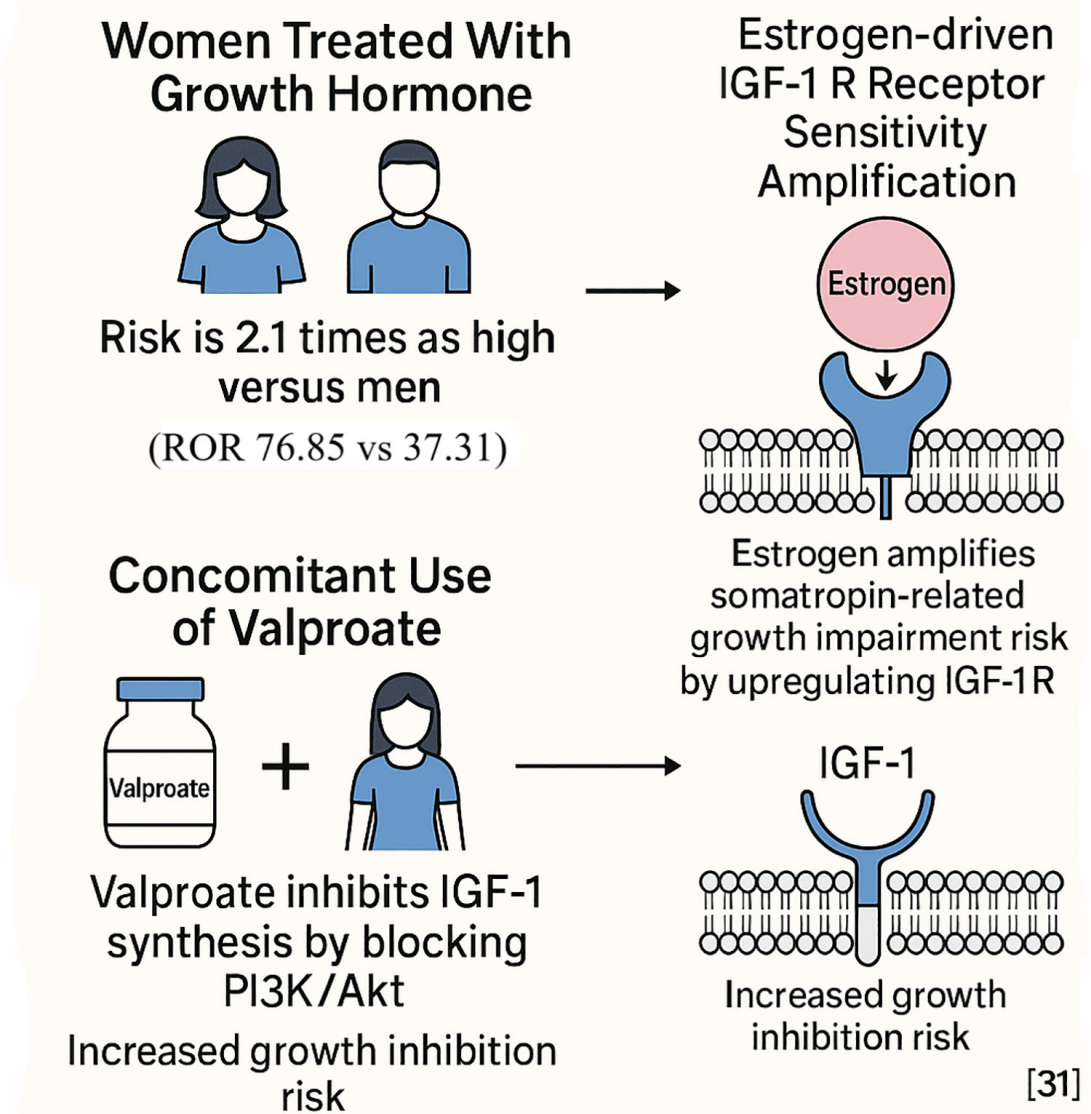
Estrogen-IGF-1R axis in female-specific growth impairment risk: a dual-hit mechanism in somatropin therapy. IGF-1 = insulin-like growth factor-1.

The sex-disaggregated signals and temporal patterns identified in this study generate hypotheses regarding potential risk-mitigation strategies that warrant prospective validation, particularly in vulnerable subgroups.^[28]^ High-risk population identification: females on somatropin carry a 2.1-fold higher risk (ROR 76.85 vs 37.31 in males), likely amplified by estrogen-driven IGF-1 receptor sensitivity (Fig. 7); adolescents ≥ 12 years on methylphenidate–corticosteroid combinations show extreme signal elevation (ROR 112.4),^[29]^ aligning with JAMA Pediatrics 2024 data on dopaminergic–glucocorticoid crosstalk-induced growth-plate senescence^[30]^; concurrent valproic acid in females warrants vigilance because valproate suppresses IGF-1 synthesis via PI3K/Akt inhibition, compounding growth-suppression risk.^[31]^

Somatropin’s 2.1-fold higher female ROR (76.85 vs 37.31) aligns with estrogen-induced down-regulation of GH receptors and accelerated epiphyseal maturation^[29,32,33]^; the male-only deflazacort signal (ROR 58.25) is consistent with androgen-driven CYP3A4 induction increasing systemic exposure.^[34]^ Concomitant methylphenidate–corticosteroid use amplifies risk through dopaminergic–glucocorticoid suppression of GH pulsatility,^[35]^ whereas TNF-α blockade may mask inflammation-related growth retardation, explaining adalimumab’s inverse association.^[36]^ Confounding factors, inherent to spontaneous-reporting systems, may modulate the interpretability of our signals and their strengths. Concomitant medications could artificially amplify signals; for instance, the co-administration of methylphenidate and corticosteroids, a common combination in clinical practice, may have inflated the observed signal strength for growth impairment due to dopaminergic–glucocorticoid crosstalk suppressing GH pulsatility. Underlying diseases served as another major confounder; baseline comorbidities like type 1 diabetes inflated corticosteroid-associated signals via hyperglycemia-induced suppression of insulin-like growth factor-1 (IGF-1), a bias only partially attenuated by methodological adjustments like inverse-probability weighting in our analysis. Most importantly, indication bias poses a non-remediable threat to causal inference in disproportionality analyses. A drug may appear protective not because it pharmacologically safeguards growth, but because it is channeled to patients whose underlying disease, once controlled, improves growth trajectories relative to the untreated or inadequately treated background population. This interpretation is strongly supported by adalimumab’s inverse association (ROR = 0.42, 95% CI 0.32–0.57), which must not be interpreted as evidence that TNF-α inhibition protects against growth impairment.

Causal inference remains constrained by indication bias and unmeasured confounders. For instance, valproic acid’s persistent signal (ROR = 14.01) could conflate epilepsy severity with direct PI3K/Akt pathway inhibition, and may necessitate Mendelian randomization to disentangle causality.^[37,38]^ Similarly, imatinib’s sex disparity (female ROR = 15.55 vs male 4.17) warrants exploration of estrogen-OCT1 transporter interactions through in vitro mechanistic studies.^[39]^ It should be emphasized that the signals identified in this study are hypothesis-generating and do not imply causality. Future frameworks must integrate causal graphical models to map confounding pathways and machine-learning-based propensity scoring to emulate randomized-trial conditions in observational pharmacovigilance data.

In conclusion, this study underscores the critical importance of sex-disaggregated analyses in pediatric pharmacovigilance, particularly for therapies impacting growth. The pronounced sex-disaggregated signals identified for drugs such as somatropin (ROR 76.85 in females vs 37.31 in males) and imatinib (ROR 15.55 in females vs 4.17 in males), alongside the male-exclusive signal for deflazacort (ROR 58.25), highlight the need for tailored monitoring and dosing strategies to mitigate risks in vulnerable subgroups. Moving forward, future research should prioritize integrating longitudinal growth data with pharmacogenomic profiles to elucidate the mechanistic roles of sex hormones, such as estrogen-driven IGF-1 receptor sensitivity or androgen-mediated CYP3A4 induction, in modulating these adverse drug reactions.^[40]^ Additionally, a critical limitation of the temporal analysis is that FAERS onset dates are frequently incomplete, imprecise, or subject to reporting delays. Reporters may estimate time-to-onset retrospectively, and missing or truncated dates can introduce right-censoring bias that artificially inflates early-failure patterns in Weibull modeling. Consequently, the observed β = 0.71 “early failure” phenotype for Growth Retardation and the wear-out pattern for Growth Failure should be interpreted cautiously as exploratory temporal hypotheses rather than precise pharmacokinetic estimates.^[41]^ Leveraging advanced predictive models, including machine learning and real-world evidence from electronic health records, wearable devices, and biobanks, could enhance early detection of high-risk patient-drug combinations. By linking FAERS data with clinical registries and embedding sex-disaggregated risk scores into decision support systems, collaborative efforts can translate these insights into actionable clinical guidelines, ultimately optimizing the benefit-risk balance of growth-modulating therapies and improving long-term outcomes for children worldwide.

## 5. Conclusion

Leveraging 3281 FAERS reports and READUS-PV-compliant methods, this exploratory study identifies disproportionate reporting signals with pronounced sex-specific patterns – most notably higher female reporting for somatropin – and delineates early-onset temporal patterns that generate testable hypotheses for future pharmacoepidemiologic investigation. These hypothesis-generating findings underscore the need for prospective, controlled studies to validate whether sex-specific monitoring protocols might improve risk stratification in pediatric growth-modulating therapy. We explicitly caution that these signals do not imply causality, incidence, or clinical efficacy of any intervention, and any translational application requires rigorous confirmation beyond spontaneous-reporting data.

## Acknowledgments

Deep gratitude is owed to the FAERS team, along with its contributing individuals.

## Author contributions

**Conceptualization:** Xu Junyu, Zhu Yan.

**Data curation:** Xu Junyu, Chen Qian, Zhu Yan.

**Formal analysis:** Xu Junyu, Xue Jingnan.

**Methodology:** Xue Jingnan.

**Project administration:** Yuan Chen.

**Software:** Qi Luying.

**Supervision:** Yuan Chen.

**Validation:** Chen Qian, Qi Luying, Yuan Chen, Zhu Yan.

**Visualization:** Xu Junyu, Xue Jingnan.

**Writing – original draft:** Xu Junyu.

**Writing – review & editing:** Chen Qian, Qi Luying, Zhu Yan.




