# Supplementary material for: Disproportionality analysis of sex-stratified adverse event signals in growth impairment: Insights from the FDA adverse event reporting system
Source: Medicine (Baltimore). 2026 Jul 17;105(29):e49802. doi: 10.1097/MD.0000000000049802 (PMC13384653; doi:10.1097/MD.0000000000049802)
Supplement: Supplementary file 1 [file medi-105-e49802-s001.docx]

***Supplementary Material***

# Sex-Disaggregated Safety Signals of Growth-Modulating Therapies in Pediatrics: A FAERS-Based Real-World Study

**Supplementary Table S1:** The 2×2 crosstab of ROR and PRR

|  | **Drug of interest** | **Other drugs** |
| --- | --- | --- |
| **AEs of interest** | DE | dE |
| **Other AEs** | De | de |

ROR, reporting odds ratio; DE, the number of interest drug reports for suspect AE; dE, the number of other drugs reports for suspect AE; De, the number of interest drug reports for other AE; de, the number of other drugs reports for other AE; PRR: Proportional Reporting Ratio.
