# Supplementary material for: Disproportionality analysis of sex-stratified adverse event signals in growth impairment: Insights from the FDA adverse event reporting system
Source: Medicine (Baltimore). 2026 Jul 17;105(29):e49802. doi: 10.1097/MD.0000000000049802 (PMC13384653; doi:10.1097/MD.0000000000049802)
Supplement: Supplementary file 2 [file medi-105-e49802-s002.docx]

***Supplementary Material***

# Sex-Disaggregated Safety Signals of Growth-Modulating Therapies in Pediatrics: A FAERS-Based Real-World Study

**Supplementary Table S2:** Summary of the main formulas used for signal detection

|  |  |
| --- | --- |
|  |  |
| **Algorithms** | **Formula** |
|  |  |
|  |  |
| ROR | $ROR=\frac{(De/de)}{(DE/dE)}$ |
| PRR | $PRR=\frac{DE/(DE+De)}{dE/(dE+de)}$ |
| SE | $SE(\ln ROR)=\sqrt{\frac{1}{De}+\frac{1}{de}+\frac{1}{DE}+\frac{1}{dE}}$ |
| 95%CI | $95\%CI=e^{\left( \ln(ROR)\pm1.96\times SE(\ln ROR) \right)}$ |
| X^2^ | $\chi^{2}=\sum\frac{(O-F)^{2}}{F},\quad O=\mathrm{DE},\quad F=\frac{(\mathrm{DE}+\mathrm{De})\cdot(\mathrm{DE}+\mathrm{dE})}{\mathrm{DE}+\mathrm{De}+\mathrm{dE}+\mathrm{de}}$ |

This table contains the mathematical formulas used to calculate the measures of association (e.g., ROR, PRR) and their corresponding 95% confidence intervals, as applied in the disproportionality analysis of this study.
